# Preparation of Fe_3_O_4_/SiO_2_/TiO_2_/CeVO_4_ Nanocomposites: Investigation of Photocatalytic Effects on Organic Pollutants, Bacterial Environments, and New Potential Therapeutic Candidate Against Cancer Cells

**DOI:** 10.3389/fphar.2020.00192

**Published:** 2020-03-04

**Authors:** Mohammad Amin Marsooli, Mahdi Rahimi-Nasrabadi, Mahdi Fasihi-Ramandi, Kourosh Adib, Mohammad Eghbali-Arani, Farhad Ahmadi, Esmail Sohouli, Ali Sobhani nasab, Seyed Ali Mirhosseini, Mohamad Reza Gangali, Hermann Ehrlich, Yvonne Joseph

**Affiliations:** ^1^Applied Microbiology Research Center, Systems Biology and Poisonings Institute, Baqiyatallah University of Medical Sciences, Tehran, Iran; ^2^Faculty of Pharmacy, Baqiyatallah University of Medical Sciences, Tehran, Iran; ^3^Department of Physics, University of Kashan, Kashan, Iran; ^4^Physiology Research Center, Iran University of Medical Sciences, Tehran, Iran; ^5^Department of Medicinal Chemistry, School of Pharmacy-International Campus, Iran University of Medical Sciences, Tehran, Iran; ^6^Young Researchers and Elite Club, Science and Research Branch, Islamic Azad University, Tehran, Iran; ^7^Social Determinants of Health (SDH) Research Center, Kashan University of Medical Sciences, Kashan, Iran; ^8^Core Research Lab, Kashan University of Medical Sciences, Kashan, Iran; ^9^Institute of Electronics and Sensor Materials, TU Bergakademie Freiberg, Freiberg, Germany

**Keywords:** Fe_3_O_4_/SiO_2_/TiO_2_, Fe_3_O_4_/SiO_2_/TiO_2_/CeVO_4_, photocatalytic, nanocomposites, photo-antibacterial, anti-cancer

## Abstract

The new nanocomposite with various molar ratios along with magnetic properties was fabricated *via* precipitation (assisted by ultrasonic) procedure. The photocatalytic effects of methylene blue (∼90% degradation for optimized sample in 100 min) for finding the optimized sample performed under visible light irradiation. Moreover, the photo-antibacterial impacts of bacteria culture environments were found with an optimized sample that had effective destruction of bacteria in comparison to control group. The cytotoxicity properties of panc1 cells and magnetic behaviors of the obtained nanomaterials were evaluated and its IC50 was about 500 mg/L. As an initial step, the structural, morphological and magnetic characteristics of the fabricated nanocomposites were evaluated by Fourier transform infrared spectroscopy (FT-IR), scanning electron microscopy (SEM), X-ray diffraction (XRD), energy dispersive X-ray (EDX) and MAP, UV-visible diffuse reflectance spectroscopy (DRS), and vibrating sample magnetometry (VSM) approaches. Based on SEM results, the size of nanoparticles in fabricated nanocomposite was nearly 50–70 nm for Fe_3_O_4_/SiO_2_/TiO_2_ and 80–100 nm for Fe_3_O_4_/SiO_2_/TiO_2_/CeVO_4_. XRD results showed that desired nanocomposites were truly synthesized without any impurities.

## Introduction

Discharge of inorganic and organic pollutants from varying industries including printing, textiles, food, and beauty products into environmental water resources has been a main environmental problem in all countries regardless of how they are developed. Up to date, different methods based on physical, chemical, and biological treatments such as membrane processes, reverse osmosis, photocatalytic degradation, photo-Fenton, ozonation, oxidation, biological, as well as electrochemical procedures have been used for remediation of industrial wastewaters (Barbusiński, 2005; Farré et al., 2005; Liu et al., 2007; Dogan and Turkdemir, 2012; Ioannou et al., 2013; Zheng et al., 2013; Suresh et al., 2014; Suresh et al., 2016; Jesudoss et al., 2017; Rajan et al., 2017; Kooshki et al., 2019; Peymani-Motlagh et al., 2019a; Peymani-Motlagh et al., 2019b; Rahimi-Nasrabadi et al., 2019; Sobhani-Nasab et al., 2019a ,Sobhani-Nasab et al., 2019b; Sobhani-Nasab et al., 2019c). Among the most commonly mentioned methods which are subject to restrictions, photocatalytic degradation systems act as a smart tool, because it is simple, highly efficient, and inexpensive (Basith et al., 2014; Vijaya et al., 2017; Sedighi et al., 2018; Eghbali-Arani et al., 2018; Sobhani-Nasab et al., 2019d). Furthermore, it has an easy operation under sunlight and ambient conditions and excellent prospects for biological and chemical sensing (Lin et al., 2012) as well as has the fewer formation of disinfection byproducts. Along with to different approaches for improving the quality of water and foods against microbial agents, utilization of nanoparticles has emerged as new horizon which could contribute to antiseptic properties of several materials such as foods and water (Zhang et al., 2018). Besides the different application of NPs, anti-cancer effects of these materials have provided attractive horizon in the treatment of several cancers. Given that the utilization of NPs are related to anti-cancer impacts against a variety of malignancies (Ezhilarasi et al., 2016; Sangsefidi et al., 2017; Ezhilarasi et al., 2018). Nowadays, some of the semiconductor nanoparticles including ZnO, Dy_2_Ti_2_O_7_, CaWO_4_, CdTiO_3_, NdVO4, and TiO_2_ have been considered as photocatalysis materials for purification of polluted waters (Zhang et al., 2000; Liu et al., 2014; Sobhani-Nasab and Sadeghi, 2016; Rahimi-Nasrabadi et al., 2016a; Rahimi-Nasrabadi et al., 2016b; Hosseinpour-Mashkani and Sobhani-Nasab, 2017). Titania (TiO_2_) as a distinctive semiconductor-based photocatalyst has been widely applied in the field of water treatment thanks to its extraordinary properties such as low cost, availability, substantial chemical stability, chemical inertness, as well as exceptional photocatalytic behavior against degradation of organic pollutants. Also, it is environmentally friendly and has an antibacterial property (Xing et al., 2014; Ide et al., 2014).

One of the most significant aspects of a photocatalyst is bacterial inactivation property. During the irradiation of a suitable light source to photocatalyst, different reactive species like superoxide and hydroxyl radicals, as well as photogenerated electron and hole, can cause microorganisms, such as bacteria and viruses which should be deactivated (Foster et al., 2011; Basith et al., 2014; Jayaprakash et al., 2015; Jesudoss et al., 2016; Jayaprakash et al., 2017). Antibacterial activity of TiO_2_ stems from the creation of reactive oxygen species (ROS) after absorption of a photon with full energy and then, excitation of a negative charge from the valence band of TiO2 to the conduction band. Superoxide anions (˙O2^-^) were formed after the transformation of an excited electron from the TiO_2_ conduction band to oxygen molecule (Carp et al., 2004). Photoexcitation process into TiO_2_ also generate holes into valance band which can oxidize some molecules such as surface absorbed H_2_O and OH into extremely reactive hydroxyl radicals (˙OH) (Kim and Choi, 2002).˙These radicals and also other ROS diffused to the solution which surrounds the photocatalyst surface can decompose organic structures and have special antibacterial effect for titanium oxide.

However, the results of previous experiments confirm that bare TiO_2_ based-nanostructure photocatalysts have weak photocatalytic performance under visible light and can experience recombination of charge carriers and a narrow light-response range, which would reasonably delay its usage in the photocatalytic operations (Kubacka et al., 2014).

To eliminate negative points about such photocatalysts, some approaches such as sensitization and combining with other semiconductors have been proposed to expand the absorption band gap of TiO_2_ and reduce the possibility of recombination electron-hole in nanostructures (Subramanian et al., 2004; Hassan et al., 2019). Some introduced composites such as Ag_3_PO_4_/CuBi_2_O_4_ (Shi et al., 2017) and, Ag/AgCl/TiO_2_ (Yu et al., 2009) exhibit high efficiency in photocatalytic degradation process compared to the one-component semiconductors because of development of heterojunction nanocomposite linking diverse semiconductors with corresponding band energy. With the formation of a suitable nanocomposite between semiconductors, the separation efficiency of electron-hole pairs generated accompanied by photon improves during the photocatalytic process.

Despite the good characteristics of the photocatalyst in water treatment, the lack of appropriate recovery of TiO_2_ nanocomposites from treated water is a major problem when it comes to applying extensively. To overcome the problem of recovering catalytic nanoparticles from water, magnetic catalysts have been focused (Maleki and Kamalzare, 2014; Maleki et al., 2015; Maleki et al., 2016a; Maleki et al., 2016b; Maleki et al., 2017; Maleki et al., 2019b; Maleki et al., 2019c; Maleki et al., 2019a; Maleki et al., 2019d). After the water treatment process, the magnetic separation of Fe_3_O_4_/TiO_2_ by using an external electromagnetic field recycles magnetic composites. The connection of Fe_3_O_4_ magnetic nanoparticles to TiO_2_ photocatalytic nanoparticles have advantages of exclusive magnetic response, chemically modifiable surface, and eco-friendly (Su et al., 2014; Maleki and Kamalzare, 2014). Also, the coating of Fe_3_O_4_ NPs with TiO_2_ prevents their massive accumulation. In addition, single Fe3O4 nanoparticles are susceptible and unstable under the reaction conditions and on the contrary, the interaction of Fe_3_O_4_ nanoparticles with TiO_2_ nanoparticles causes recombination of electrons and holes and, consequently, decrease photocatalytic properties (Absalan and Nikazar, 2016). Incorporation of a layer such as a controlled silicon oxide layer between the magnetic core and photocatalyst shell can decline the negative effect of Iron oxide on the photocatalysis process of titanium oxide, retain magnetic properties, give protection Fe_3_O_4_ against oxidation, and enrich the removal efficiency. Recently, a number of research has been done on the construction of recyclable photocatalytic nanocomposites of Fe_3_O_4_/SiO_2_/TiO_2_ with core–shell structure (Rashid et al., 2015). On the other hand, the malignant growth of pancreas is one of the most important causes of cancer-related death. Despite the fact that the prevalence of pancreatic cancer is much less than the breast or bowel tumor, nearly 3% of patients remain alive over five years, whereas the normal life expectancy is below 6 months (Kowalski et al., 2018).

In this study, our research group managed to synthesize Fe_3_O_4_/SiO_2_/TiO_2_/CeVO_4_ (Iron oxide/silicon oxide/titanium oxide/cerium oxide) nanocomposites, and combined the advantages of heterogeneous catalysis, recycling of nanocomposite, antibacterial, and cytotoxicity activity, and enhancing of the photocatalytic property of TiO_2_ photocatalyst to degrade impurities.

## Experimental

### Characterization

The morphology and size in the preparation of Fe_3_O_4_/SiO_2_/TiO_2_/CeVO_4_ were accomplished through using FESEM. Fe_3_O_4_/SiO_2_/TiO_2_/CeVO_4_ sonicated for 10 min. Then, 10 μL of sonicated Fe_3_O_4_/SiO_2_/TiO_2_/CeVO_4_ was dropped on a copper grid and imaging was accomplished at an accelerating voltage of 200 kV. For the elemental analysis of the Fe_3_O_4_/SiO_2_/TiO_2_/CeVO_4_, Energy-dispersive X-ray spectroscopy (EDX) was accomplished during SEM imaging. XRD analyses were conducted using a Philips X'pert Pro MPD with a graphite-filtered Cu Kα (k = 0.154 nm) radiation. These analyses were accomplished in a 2θ window ranging from 10° to 90°, at a 0.021 step size and 0.9 s per point measuring time. The FTIR analysis of Fe_3_O_4_/SiO_2_/TiO_2_/CeVO_4_ was acquired using a Nicolet Magna- 550 spectrometer and KBr pellets with scan speed of 65 spectra/s at 16 cm^−^. Thermal degradation or thermal stability study of Fe_3_O_4_/SiO_2_/TiO_2_/CeVO_4_ were done by employing a TGA instrument with a flow rate of 30.0 ml min °C (Shimadzu TGA-50H). The surface area in characterization of the Fe_3_O_4_/SiO_2_/TiO_2_/CeVO_4_ was accomplished on Brunauer-Emmett- Teller (BET) surface area analyzer.

### Preparation of Fe_3_O_4_ Nanoparticles

This synthesis is based on previous work (Wei et al., 2012). The reagents of analytic grade (FeCl_3_·6H_2_O, FeCl_2_·4H_2_O, and NaOH) were used as a precursor. Generally, FeCl_3_·6H_2_O and FeCl_2_·4H_2_O with 1:2 molar ratio was dissolved in deionized water at 80 °C, and then NaOH solution (3 mol. L^-1^) was added into the above mentioned solution dropwise under constant mechanical stirring for half an hour to reach final pH of 11. The precipitate was stirred at 80°C for 30 min, and cooled in normal temperature. Following the separation of resulted particles by a magnet, it washed frequently with deionized water and ethanol until pH of 7 was achieved. The Fe_3_O_4_ nanoparticles were dried at 60°C in vacuum for 8 h.

### Preparation of Fe_3_O_4_/SiO_2_ Nanocomposite

The iron oxide/silicon oxide nanocomposite was achieved by modifying the Stober process *via* the hydrolysis of tetraethyl orthosilicate (TEOS) in the presence of Fe_3_O_4_ nanoparticles based on the previous work (Wei et al., 2012; Abbas et al., 2014). We dispersed 0.1 g of as-synthesized Fe_3_O_4_ in 20 ml of water by using an ultrasonic wave. Subsequently, 2.5 ml of aqueous ammonia solution (28%) and 80 ml of ethanol were added to the mixture. Subsequent, 0.35 ml of TEOS was added dropwise into the mixed Fe_3_O_4_ nanoparticles under ultrasonic irradiation at room temperature. The stirring continued for half day. The separation of obtained precipitate was achieved by an external magnet and washed with water several times. Eventually, the collected precipitate was, once again, washed with water and dried in a vacuum oven at 60°C for 6 h.

### Preparation of Fe_3_O_4_/SiO_2_/TiO_2_ Nanocomposite

0.9 g of two-component iron oxide/silicon oxide nanocomposite were dispersed *via* an ultrasonic bath in 150 ml of 2-propanol for 15 min. Afterward, we added 8 ml of PEG 400 into the mixture. In the next vessel, 5 ml of tetra normal buthyl titanate (TNBT) was added to 20 ml of 12 ml of 2-propanol and 1 ml acetylacetone and stirred on a magnetic stirrer for 10 min. The second vessel was added step by step to the first mixture while it was stirring by mechanical stirrer. After 20 min, 3 ml of deionized water was added and the final mixture stirred at 70°C for 12 h. The gray precipitate of oxide/silicon oxide/titanium oxide was separated by an external magnet and washed thoroughly with ethanol and deionized water prior to drying at 60°C for 6 h and then was calcinated at 450°C for 3 h (N_1_).

### Preparation of Fe_3_O_4_/SiO_2_/TiO_2_/CeVO_4_ Nanocomposite

To prepare the Fe_3_O_4_/SiO_2_/TiO_2_/CeVO_4_ nanocomposite with 1:1:1:1 molar ratio of CeVO_4_ to the previous phases, in the first container 0.371 g of non-calcinated three parts of nanocomposite, Fe_3_O_4_/SiO_2_/TiO_2_, were dispersed in 100 ml of distilled water by ultrasonic waves. Then, 0.117 g of NH_4_VO_3_ added to the mixture. In the second container, 0.434 g of Ce(NO_3_)_3_.6H_2_O was dissolved in 50 ml of water. The first mixture was subjected to ultrasonic waves by a probe (400 W), and the solution of the second container was added as a dropwise to it. The reaction continued for 15 min and finally, the precipitate was washed repeatedly with deionized water as well as ethanol and dried at 60°C for 4 h. For calcination of the obtained sediments, it was placed at 450°C for 3 h (N_3_). To prepare sediments with molar ratios of 1:1:1:0.5 (N_2_), 1:1:1:1.5 (N_4_), and 1:1:1:2 (N_5_), 0.217g of Ce(NO_3_)_3_.6H_2_O and 0.06g of NH_4_VO_3_, 0.65g of Ce(NO_3_)_3_.6H_2_O and 0.176g of NH_4_VO_3_, and 0.87g of Ce(NO_3_)_3_.6H_2_O and 0.234g of NH_4_VO_3_ were used according to the same procedure, respectively.

The reaction mechanism the Fe_3_O_4_/SiO_2_/TiO_2_/CeVO_4_ nanoparticles can be proposed as following:

(1) $$2{\text{FeCl}}_{3}.9{\text{H}}_{2}\text{O}+{\text{H}}_{2}\text{O}\to \;2{\text{Fe}}^{3+}\;+\;18{\text{Cl}}^{-}\;+\;19{\text{H}}_{2}\text{O}$$

(2) $${\text{FeCl}}_{2}.9{\text{H}}_{2}\text{O}+{\text{H}}_{2}\text{O}\to {\text{ Fe}}^{2+}\;+\;6{\text{Cl}}^{-}\;+\;10{\text{H}}_{2}\text{O}$$

(3) $${\text{Fe}}^{2+}+\;2{\text{Fe}}^{3+}+8\text{NaOH}+6{\text{Cl}}^{-}+{\text{H}}_{2}\text{O}\to \text{ Fe}{\left(\text{OH}\right)}_{2}\;+\;2\text{Fe}{\left(\text{OH}\right)}_{3}+\text{ NaCl}$$

(4) $$\text{Fe}{\left(\text{OH}\right)}_{2}\;+\;2\text{Fe}{\left(\text{OH}\right)}_{3}+\;\Delta \text{T}\to {\text{ Fe}}_{3}{\text{O}}_{4}+\;4{\text{H}}_{2}\text{O}$$

(5) $${\text{H}}_{2}\text{O}+\text{Si}{\left({\text{OC}}_{2}{\text{H}}_{5}\right)}_{4}\to {\text{ C}}_{2}{\text{H}}_{5}\text{OH}+\text{ Si}{\left(\text{OH}\right)}_{4}$$

(6) $$\text{Si}{\left(\text{OH}\right)}_{4}+\text{Si}{\left(\text{OH}\right)}_{4}\to {\left(\text{OH}\right)}_{3}\text{Si}-\text{O}-\text{Si}{\left(\text{OH}\right)}_{3}+{\text{ H}}_{2}\text{O}$$

(7) $${\left(\text{OH}\right)}_{3}\text{Si}-\text{O}-\text{Si}{\left(\text{OH}\right)}_{3}+\;\Delta \text{T}\to {\text{SiO}}_{2}+{\text{ H}}_{2}\text{O}$$

(8) $${\text{H}}_{2}\text{O}+\text{Ti}{\left({\text{OC}}_{4}{\text{H}}_{9}\right)}_{4}\to {\text{ C}}_{4}{\text{H}}_{9}\text{OH}+\text{ Ti}{\left(\text{OH}\right)}_{4}$$

(9) $$\text{Ti}{\left(\text{OH}\right)}_{4}+\text{Ti}{\left(\text{OH}\right)}_{4}\to {\left(\text{OH}\right)}_{3}\text{Ti}-\text{O}-\text{Ti}{\left(\text{OH}\right)}_{3}+{\text{ H}}_{2}\text{O}$$

(9) $${\left(\text{OH}\right)}_{3}\text{Ti}-\text{O}-\text{Ti}{\left(\text{OH}\right)}_{3}+\;\Delta \text{T}\to {\text{TiO}}_{2}+{\text{ H}}_{2}\text{O}$$

(10) $$\text{Ce}{\left({\text{NO}}_{3}\right)}_{3}.6{\text{H}}_{2}\text{O}+{\text{H}}_{2}\text{O}\to {\text{ Ce}}^{3+}\;+\;3{\text{NO}}_{3}^{-}\;+\;7{\text{H}}_{2}\text{O}$$

(11) $${\text{NH}}_{4}{\text{VO}}_{3}\;+{\text{ H}}_{2}\text{O }\to {\text{NH}}_{4}^{+}\;+{\text{VO}}_{3}^{-}\;+{\text{ H}}^{+}\;+{\text{ OH}}^{-}$$

(12) $${\text{Ce}}^{3+}+{\text{VO}}_{3}^{-}\;\to {\text{ CeVO}}_{4}$$

(13) $$\text{Fe}{\left(\text{N}{\text{O}}_{3}\right)}_{3}\;.9{\text{H}}_{2}\text{O}+\text{Fe}{\left(\text{N}{\text{O}}_{3}\right)}_{2}\;9{\text{H}}_{2}\text{O}+\text{NaOH}+\text{Si}{\left(\text{O}{\text{C}}_{2}{\text{H}}_{5}\right)}_{4}+\text{Ti}{\left(\text{O}{\text{C}}_{4}{\text{H}}_{9}\right)}_{4}+\text{Ce}{\left(\text{N}{\text{O}}_{3}\right)}_{3}\;6{\text{H}}_{2}\text{O}+3\text{N}{\text{H}}_{4}\text{V}{\text{O}}_{3}\to \text{N}{\text{H}}_{4}\text{N}{\text{O}}_{3}+{\text{C}}_{2}{\text{H}}_{5}\text{OH}+\text{NaN}{\text{O}}_{3}+{\text{C}}_{4}{\text{H}}_{9}\text{OH}+\text{F}{\text{e}}_{3}{\text{O}}_{4}/\text{Si}{\text{O}}_{2}/\text{Ti}{\text{O}}_{2}/\text{CeV}{\text{O}}_{4}+{\text{H}}_{2}\text{O}$$

### Photocatalytic Evaluation

In order to find the optimal sample, the photocatalytic test was performed under the visible light (assisted by H_2_O_2_) with 20 ppm of MB. For each test 200 mg/L of the photocatalyst was added into 0.3 L of 20 ppm MB solution. To reach improved efficiency 1 ml/100 ml of 25% H2O2 was added to the reaction photoreactor. In order to reach an adsorption/desorption equilibrium between catalysts and MB solution, the photoreactor container was stirred in a dark condition for 20 min prior to being subjected to the visible light source (250 W xenon lamp). Then, 4 ml of the solution were taken by a pipette and placed in dark and under light for every 10 and 20 min, respectively. Afterward, in order to separate the catalyst, it was centrifuged at 5,000 rpm for 5 min. We, eventually, evaluated the concentration of MB through a UV-vis spectrometer to reach the outcome of photodegradation.

To analyze hydroxyl radicals which have been generated in the photocatalyst/water interface, we used the photoluminescence technique. In this procedure terephthalic acid (TA) was considered as a probe. As a result, we managed to produce 2-hydroxyterephthalic acid with highly intense fluorescence. In fact, it is the product of the TA method accompanied by hydroxyl radicals. In other words, the intensity of fluorescence is positively correlated with concentration of produced hydroxyl radicals. The set up for this test is similar to photocatalytic experiment under ultraviolet irradiation.

We prepared the reaction suspension by means of adding 0.03 g of photocatalyst (0.1 g/L) into the 300 ml aqueous solution of terephthalic acid and NaOH. The earlier and former acids were prepared with a concentration of 0.0005 M (0.451 g in 500 ml distilled water) and 0.002 M (0.04 g in 500 ml distilled water), respectively.

We utilized trapping tests of superoxide radical (O_2_^−^), holes, as well as hydroxyl radical (OH), *via* making use of, benzoquinone, citric acid, and tert-butanol, respectively, to determine the principal oxidative species in the photocatalytic procedure. Therefore, we prepared 300 ml of MB with 20 ppm and add 3 mmol from one of the scavengers into the solution. Subsequently, we dispersed 0.03 g of photocatalyst and added it to the abovementioned solution. Afterward, we subjected our system to irradiation of UV, pipetted 4 ml of solution each 10 min, and separate catalyst from contaminant *via* centrifuging. We utilized a UV-spectrophotometer to monitor making process of each reaction.

To find out the effectiveness of synthesized materials to photocatalytically decolorize bacteria, Gram-positive *Staphylococcus aureus* (ATCC 6538), and Gram-negative *Escherichiacoli* (ATCC 25922) were selected to carry out pure culture investigation. First of all, we inoculated sterile 5 ml aliquots of Mueller Hinton broth (Merck) with *E. coli* and *S. aureus* and incubated overnight at 37°C. Next, we centrifuged them, washed with phosphate buffered saline (PBS) and adjusted appropriately their concentration. In order to evaluate the impact of Fe_3_O_4_/SiO_2_/TiO_2_ (N_1_) and Fe_3_O_4_/SiO_2_/TiO_2_/CeVO_4_ (N_5_) on the chosen bacteria, 2% (w/w) suspensions of nanocomposites in Mueller Hinton (M-H) broth was prepared. The effect of Fe_3_O_4_/SiO_2_/TiO_2_ (N_1_) and Fe_3_O_4_/SiO_2_/TiO_2_/CeVO_4_ (N_5_) nanocomposites on the behavior of *E. coli* and *S. aureus* was obtained by evaluating the growth of the cultures with photocatalysts compared to the growth of the culture in the medium. Mueller Hinton broth inoculated with bacteria only functioned as a positive control. For the test, sterile microtubes were filled with 0.5 ml of previously prepared solutions 1 mg. ml^-1^ final concentration. Next, bacterial suspensions were added to each microtube (approximately 10^5^ colony-forming units/ml). The prepared systems were divided into three sets: we exposed the first set to the source of UV light (50W) for a period of 16 min and samples were taken at 0, 2, 4, 8, and 16 min. The second set was subjected to the source of visible light (Xe lamp) and we left the final set with no access to light for a total period of 160 min. For evaluation, samples from the second and third groups were taken at 0, 20, 40, 80, and 160 min. The serial dilutions in PBS were prepared with achieved bacterial culture aliquots and the number of viable cells (CFU/ml) was assessed using the pour plate technique. All experiments were tested in triplicate.

### Cell Culture

We prepared Panc1 cell lines from the National Cell Bank of Iran (NCBI, Tehran) and grew it in the RPMI 1640 medium (Gibco) added to 10% (v/v) Fetal Bovine Serum (FBS), penicillin/streptomycin (100 IU/ml, 100 μg/ml respectively) (Sigma, Germany). We incubated and kept cells in an atmosphere which has been humidified at 37°C and 5% CO_2_. As soon as we reached 85% confluence, we rinsed cells with pure RPMI and gathered them *via* 0.25% Trypsin/EDTA solution (Sigma, Germany). All experiments were conducted three times All experimental protocols were approved by the Baqiyatallah University of Medical Sciences Ethical Committee by letter IR.BMSU.REC.1397.409.

### MTT Assay

The cytotoxicity effects of Fe_3_O_4_/SiO_2_/TiO_2_/CeVO_4_ nanocomposite on Panc1 cells was evaluated using the MTT assay. This procedure is based on the potential of feasible cells to generate blue formazan crystals from yellow tetrazolium salt MTT *via* mitochondrial dehydrogenase. We positioned gathered cells in a plate with 96 cells (Nunc, Denmark) and density of 10^4^ cells/well. Next, we selected cells with varied concentrations of nanocomposites (2, 1, 0.5, 0.25, 0.125, 0.063, 0.0315, 0.0157 mg/ml), and incubated the microplate at 37°C and 5% CO_2_ for one and two days. Afterward, we added 10 µl of MTT reagent to each well and incubated the plate for 4h. Next procedure was discarding of supernatants, adding 100 µl of the DMSO into each well, and incubating plates for 20 min, in sequence. Eventually, we managed to monitor cytotoxicity *via* evaluating the absorbance at an appropriate wavelength (ƛ = 570 nm) making use of ELISA plate reader (Lab System). The cell cytotoxicity and viability were computed, in terms of percentage, based on the following formula (Doosti et al., 2018):

(14) % Cytotoxicity = 1 − average absorbance of toxicant/average absorbance of negative control×100

## Results and Discussion

### Characterization

**Figure 1:**
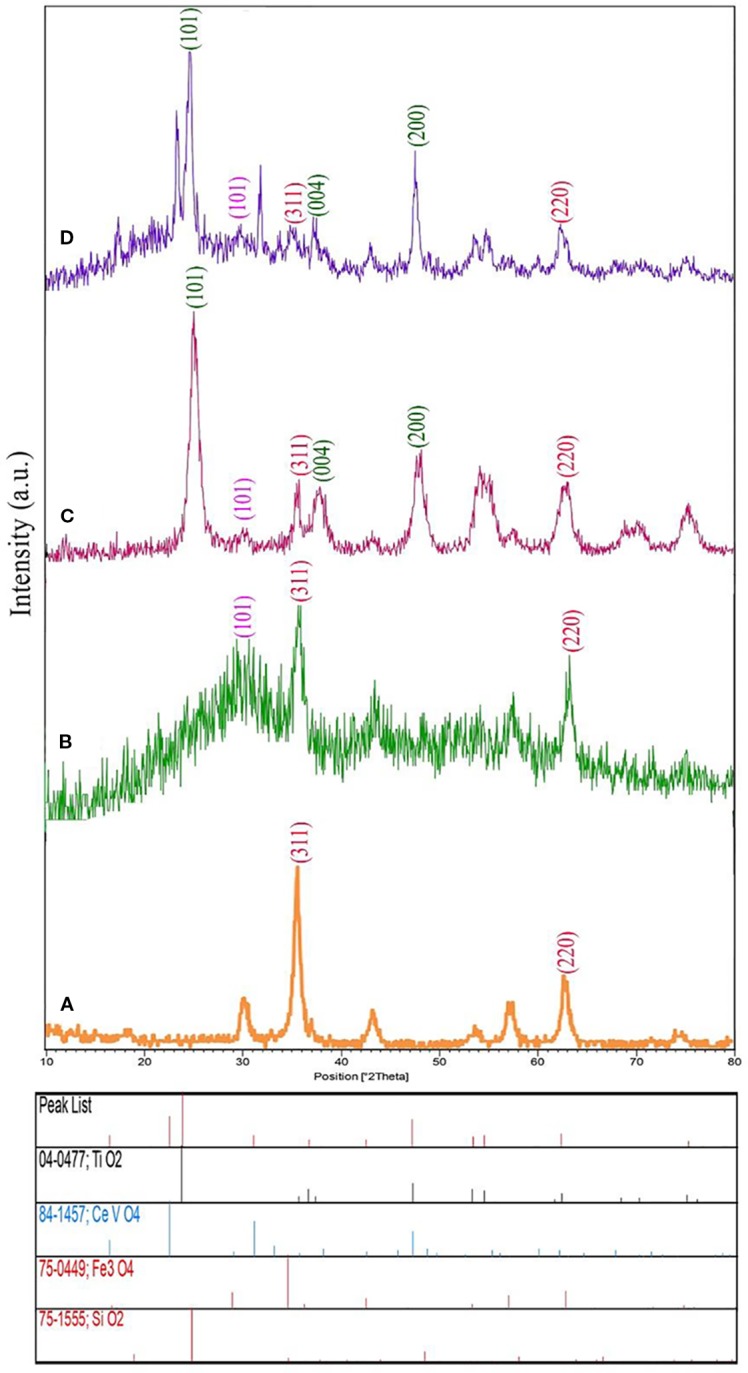
X-ray diffraction (XRD) analysis of **(A)** Fe_3_O_4_ nanoparticles, **(B)** Fe_3_O_4_/SiO_2_, and **(C)** Fe_3_O_4_/SiO_2_/TiO_2_ (N_1_), and **(D)** Fe_3_O_4_/SiO_2_/TiO_2_/CeVO_4_ (N_5_) nanocomposites.

The XRD plot of the Fe_3_O_4_ nanosized with pure phase cubic and space group of Fd-3m is revealed in **Figure 1A**. In this figure, there are a series of diffraction peaks at 35.60° ((311) line), and 63.10° ((220) line), which is in good agreement with Fe_3_O_4_ (JCPDS75-0449) and the calculated cell parameters of a = b= c= 8.3200 Å [5]. The XRD plot of the Fe_3_O_4_/SiO_2_ nanostructure has appeared in **Figure 1B**. The Fe_3_O_4_/SiO_2_ nanostructures are composed of two pure phases including Fe_3_O_4_ (JCPDS75-0449 and space group Fd-3m) as well as SiO_2_ (JCPDS 75-1555 and space group P6222) that show a series of diffraction peaks at the position of 26.13° with lines (101) which is well-matched with pure phase hexagonal Fe_3_O_4_ nanostructure. Besides, the XRD plot for Fe_3_O_4_/SiO_2_ nanostructure prepared in room temperature with low crystallinity has been displayed in **Figure 1B** and the XRD plot of the Fe_3_O_4_/SiO_2_/TiO_2_ nanostructure have been shown in **Figure 1C**. The Fe_3_O_4_/SiO_2_/TiO_2_ nanostructures contain three pure phases such as Fe_3_O_4_ (JCPDS75-0449 and space group Fd-3m), SiO_2_ (JCPDS 75-1555 and space group P6222) and TiO_2_ (JCPDS 04-0477 and space group I41/amd) which indicate a series of diffraction peaks at the position of 25.32°, 27.52°, and 48.00° with lines (101), (004), and (200) which is compatible with pure phase of tetragonal TiO_2_ nanostructures.

The XRD graph of the Fe_3_O_4_/SiO_2_/TiO_2_/CeVO_4_ nanostructure has been demonstrated in **Figure 1D**. The Fe_3_O_4_/SiO_2_/TiO_2_/CeVO_4_ nanostructures involve four pure phases like Fe_3_O_4_ (JCPDS75-0449 and space group Fd-3m), SiO_2_ (JCPDS 75-1555 and space group P6222), TiO_2_ (JCPDS 04-0477 and space group I41/amd), as well as CeVO_4_ (JCPDS 084-1457 and space group I41/amd) suggesting a series of diffraction peaks at 24.03° Line (200), 32.04° Line (112) and 47.86° Line (312). One can simply find the good consistency between pure phases of tetragonal CeVO_4_ nanostructure. Also, adding the CeVO_4_ layer can result in decrease of intensity of three previous parts and therefore, the crystal size of nanocomposites have been increased.

**Figure 2:**
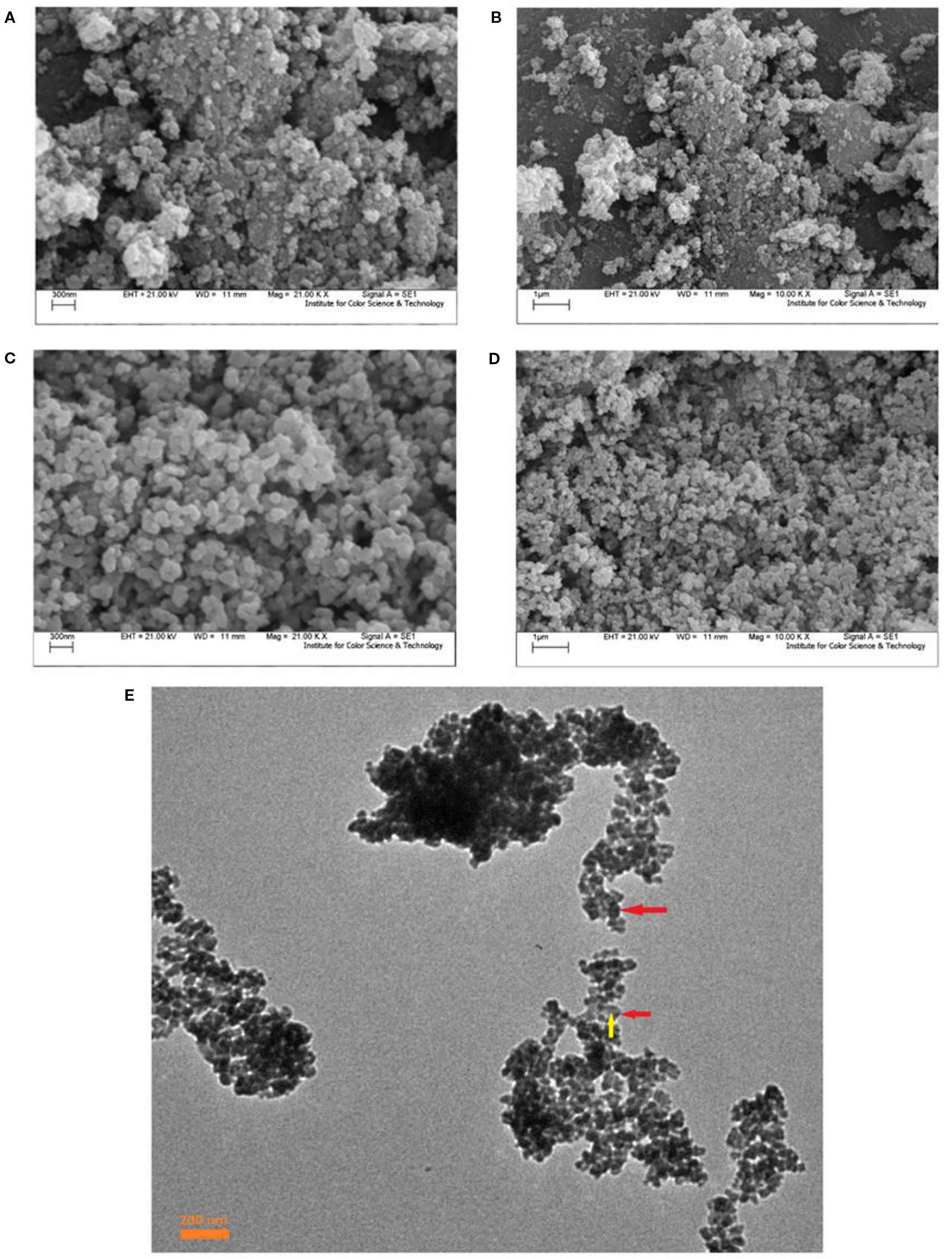
Scanning electron microscopy (SEM) images of prepared nanocomposites; Fe_3_O_4_/SiO_2_/TiO_2_ (N_1_) **(A, B)** Fe_3_O_4_/SiO_2_/TiO_2_/CeVO_4_ (N_5_); **(C, D)**, TEM image of N_5_ sample **(E)**.

**Figure 2** shows the SEM images of synthesized Fe_3_O_4_/SiO_2_/TiO_2_ (N1) and optimum sample of Fe_3_O_4_/SiO_2_/TiO_2_/CeVO_4_ (N5) nanoparticles under low and high magnification. The Fe_3_O_4_/SiO_2_/TiO_2_ nanoparticles with diameters of 50–70 nm and Fe_3_O_4_/SiO_2_/TiO_2_/CeVO_4_ (N5) nanoparticles with diameters of 80–100 nm were found to be well synthesized. Morphologically, both of them have the same shape and uniform distribution. As well, TEM image of N_5_ sample (**Figure 2E**) was taken where the darker regions shown in the photograph are related to the magnetic component and the brighter regions are related to the rest of the phases forming this nanostructure.

**Figure 3:**
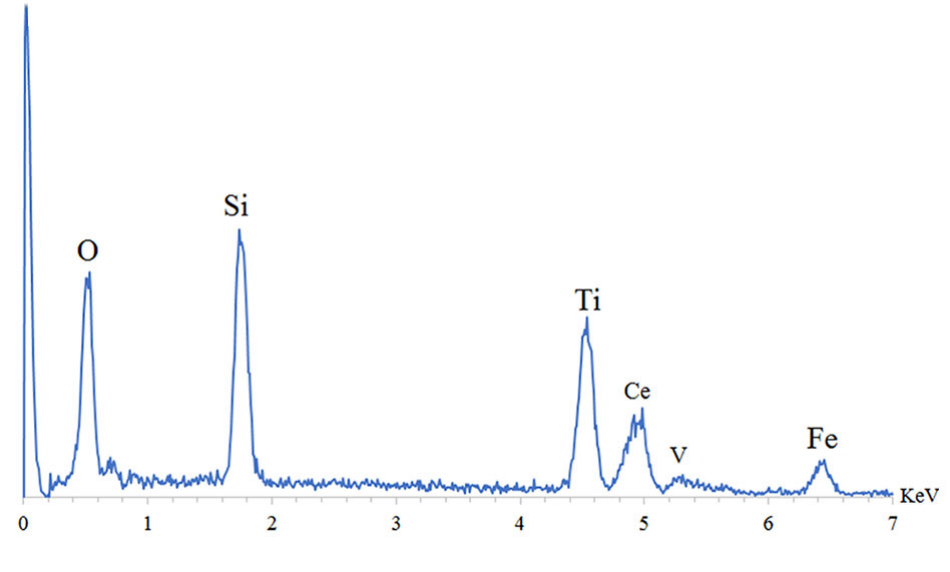
Energy-dispersive X-ray spectroscopy (EDS) spectra of optimum sample Fe_3_O_4_/SiO_2_/TiO_2_/CeVO_4_ (N_5_).

**Figure 4:**
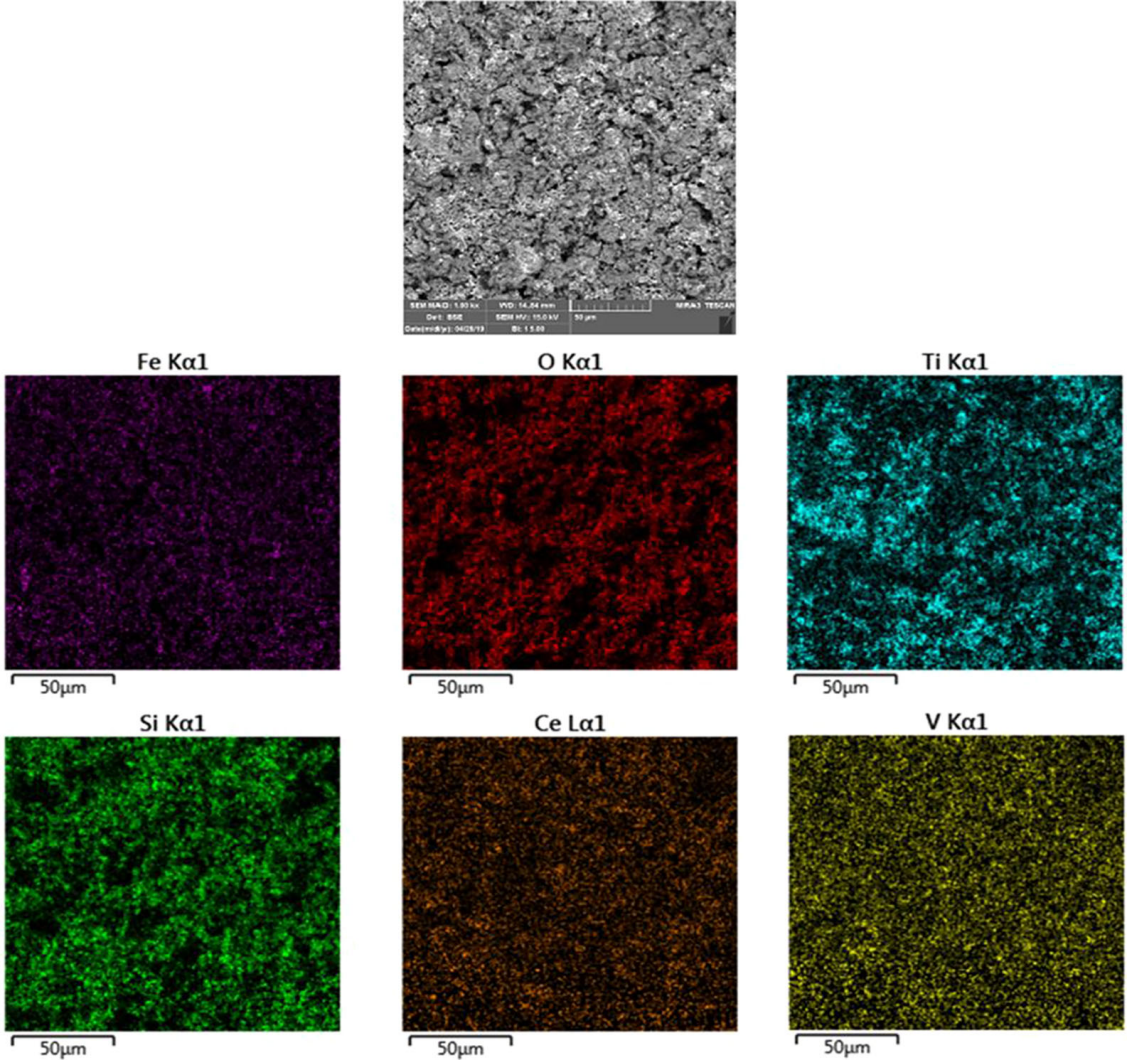
MAP analysis of Fe_3_O_4_/SiO_2_/TiO_2_/CeVO_4_ (N_5_) nanocomposite.

**Figure 5:**
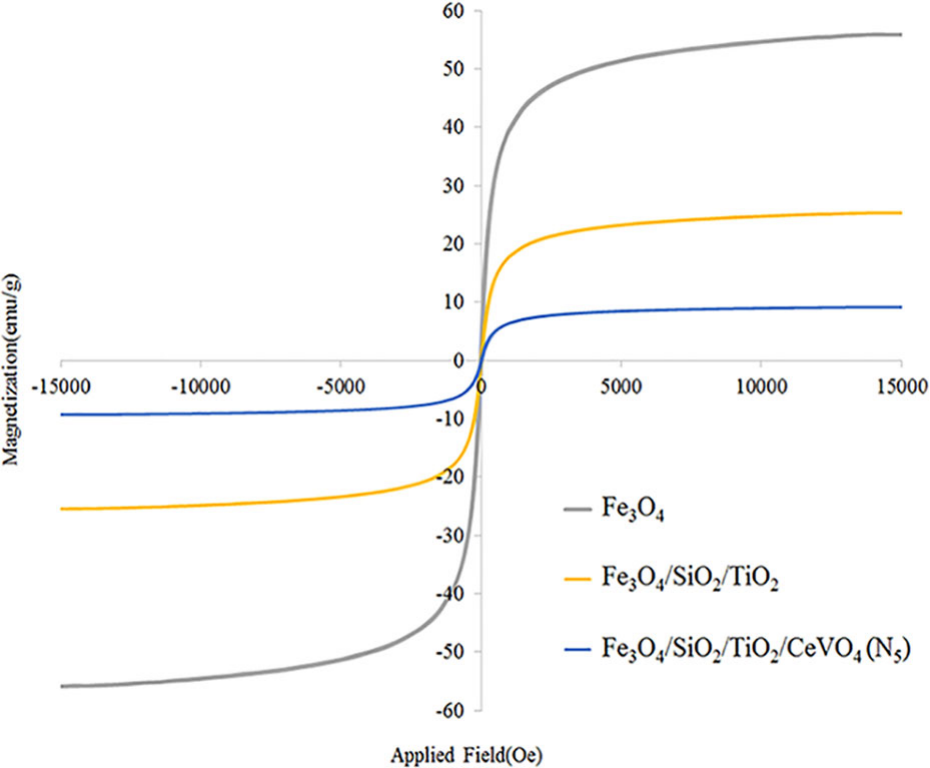
Room temperature magnetization curve of Fe3O4 nanoparticles, Fe3O4/SiO2/TiO2 (N_5_), and Fe_3_O_4_/SiO_2_/TiO_2_/CeVO_4_ (N_5_) nanocomposites.

Also, investigate the phase of the optimum synthesized nanocomposite (N5), we further characterized the elements of synthesized nanocomposite using energy-dispersive X-ray spectroscopy (EDS) analyses. The EDS analysis confirmed the presence of the desired elements in the nanocomposite, as shown in **Figure 3**. To investigate the uniformity of the nanoparticles distribution, an elemental mapping analysis was conducted with EDS, as shown in **Figure 4**. The magnetic properties of the magnetic nanocomposites were measured using a vibrating sample magnetometer (VSM). **Figure 5** shows the room temperature magnetization curves of simple Fe_3_O_4_ nanoparticles, Fe_3_O_4_/SiO_2_/TiO_2_ nanocomposite (N_1_) and the Fe_3_O_4_/SiO_2_/TiO_2_/CeVO_4_ (N_5_) samples. As illustrated in this figure, the saturation magnetizations (Ms) of the samples are 55.7, 25.3, and 9.2emug^-1^, respectively. Our outcomes for the VSM analysis prove that the synthesized photocatalysts show typical superparamagnetic behavior. The reduction in the measured saturation magnetization is owing to the added next nanoparticles layers on Fe_3_O_4_.

**Figure 6:**
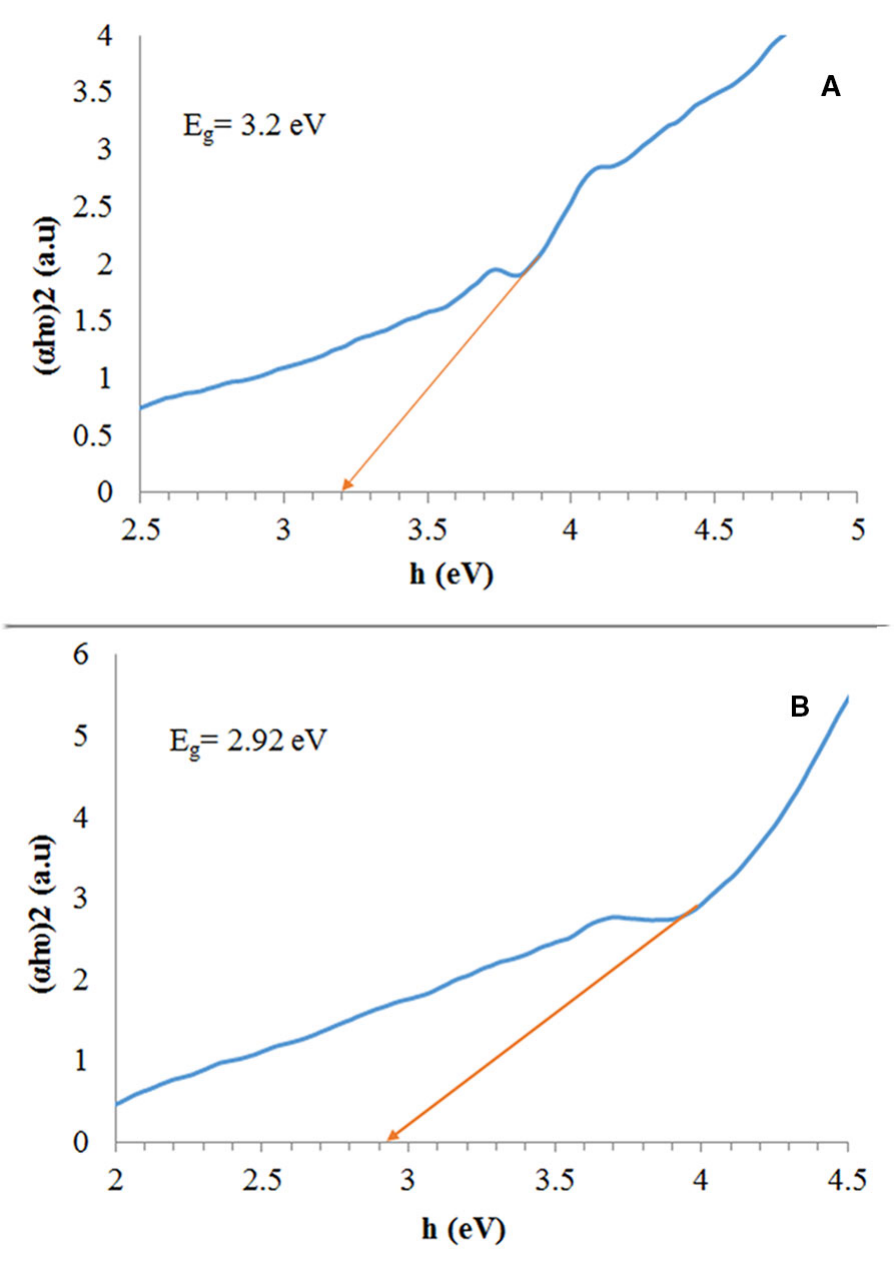
Plot of ${\left(\alpha h\upsilon \right)}^{\frac{1}{2}}$vs. energy (*hυ*) of N_1_ sample **(A)** and N_5_ sample **(B)**.

We calculated the band gap based on a Tauc plot, a technique which is extensively utilized to obtain band gaps which have been shown in **Figure 6**. The estimated band gap values (Eg) were 3.2, and 2.92 eV for the Fe_3_O_4_/SiO_2_/TiO_2_, and Fe_3_O_4_/SiO_2_/TiO_2_/CeVO_4_ (N_5_) samples, respectively.

**Figure 7:**
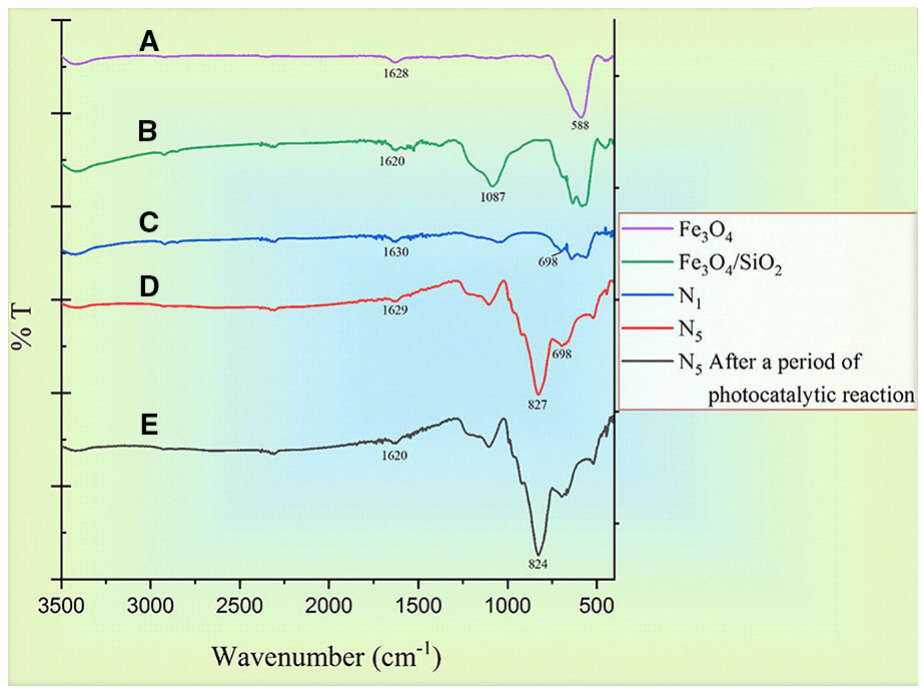
FTIR spectrums of **(A)** Fe_3_O_4_, **(B)** Fe_3_O_4_/SiO_4_ sample, **(C)** Fe_3_O_4_/SiO_2/_TiO_2_ (N_1_), **(D)** Fe_3_O_4_/SiO_2/_TiO_2_/CeVO_4_ (N_5_) sample, and **(E)** N_5_ after a period of photodegradation of MB.

**Figure 7** shows the FTIR spectrum of Fe_3_O_4_ (a), Fe_3_O_4_/SiO_2_ (b), Fe_3_O_4_/SiO_2/_TiO_2_ (N_1_) (c), Fe_3_O_4_/SiO_2/_TiO_2_/CeVO_4_ (N_5_)(d), as well as N_5_ sample after a complete photocatalytic reaction period with 20 ppm of MB dye. At all spectrums, the presence of water is shown by the appearance of the bending mode at around ∼1,600 cm^-1^ and the stretching mode at around ∼3,300 cm^-1^ (Kunarti et al., 2016; Doosti et al., 2018). A strong peak at ∼588 cm^−1^ of Fe_3_O_4_ was obtained which was assigned to the Fe-O stretching vibration. There was a new strong band around 1,087 cm^−1^ in **Figure 7B** that came from the Si-O bond in SiO_2_(Kunarti et al., 2016) and the vibration band for the fingerprint of Ti–O–Ti bond in **Figure 6C**, which is located around 700 cm^-1^ (Kunarti et al., 2016; Doosti et al., 2018). The presence of a strong peak at 827 cm^-1^ is due to the V–O stretching vibration of VO_4_ which has been displayed in **Figure 7D** (Marsooli et al., 2019). **Figure 7E** is related to the N_5_ sample after a photocatalytic reaction period, which is in contrast to **Figure 7D**. Clearly, it has not changed to much extent and is slightly noisy, suggesting the synthesized nanocomposites did not absorb colored materials and have not been destroyed.

### Photocatalytic Evaluation

**Figure 8:**
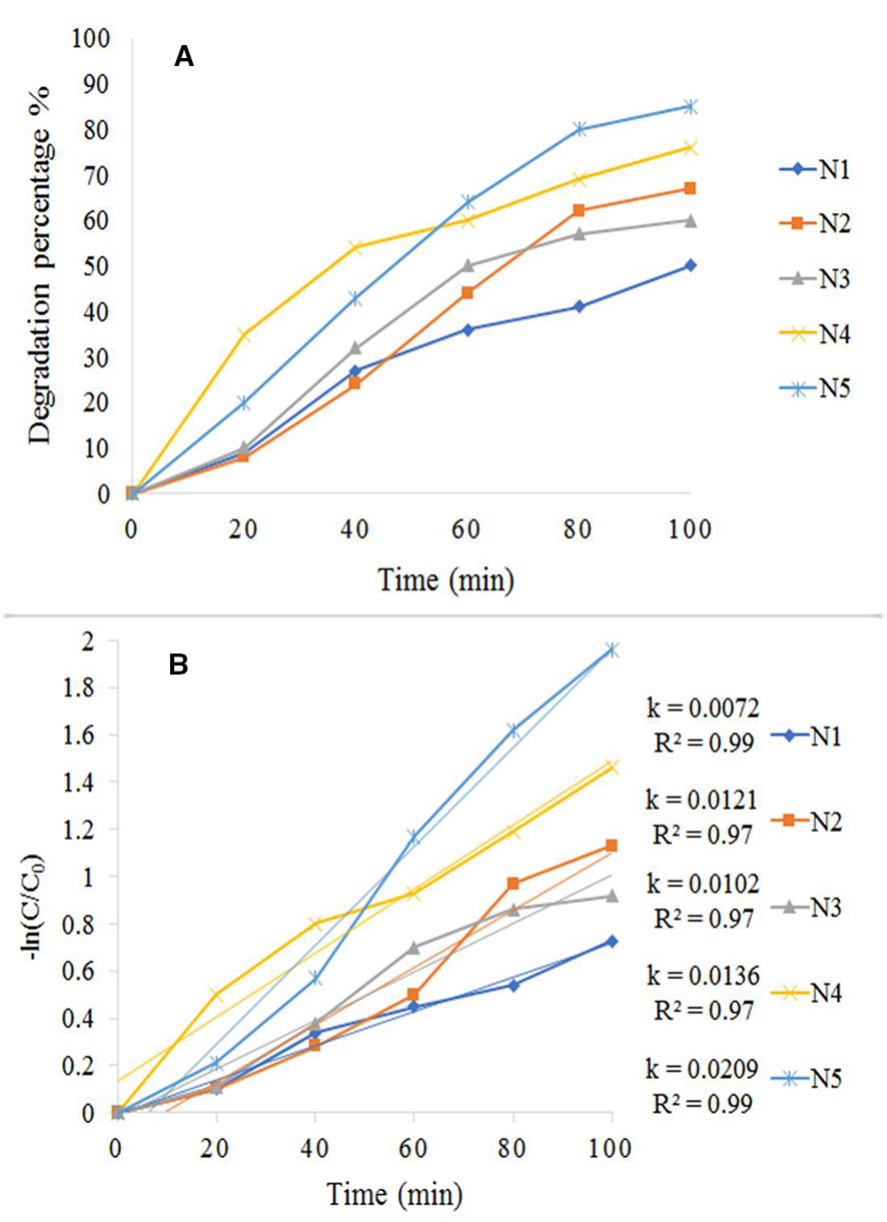
**(A)** Photocatalytic degradation rate of MB, **(B)** Kinetic fit plot of -ln (C/Co) vs. time for different synthesized photocatalyst.

The result of a photocatalytic test under the visible spectrum is shown in **Figure 8**. We conducted this test for all synthesized samples (N_1_-N_5_) by 20 ppm of MB (assisted by 1 ml/100 ml H_2_O_2_). Among all the tests performed within 100 min, it was found that the photocatalytic influence of N_5_ sample (1:1.5 molar ratio) was higher than the rest of the specimens (**Figure 8A**). **Figure 8B** shows the Kinetic fit plot of -ln (C/Co) vs. time for different synthesized photocatalysts to be pseudo-first order. The slope of the linear regression was utilized as the first order reaction rate constant. Eventually, N_5_ sample showed the best performance and as a result, could be considered as a good candidate for the destruction of pollutants from the water. We have shown the efficiency of magnetic photocatalysts under visible and ultraviolet light in **Table 1**. According to the table, the degradation of organic dyes takes more time under visible light because the wavelengths of visible light have low energy. However, in this study, fabrication of Fe_3_O_4_/SiO_2/_TiO_2_/CeVO_4_ nanocomposite for the first time, it was found that they have higher photocatalyst efficiency rather than other magnetic photocatalysts under visible light.

**Table 1:** Characterization comparison of Fe_3_O_4_/SiO_2/_TiO_2_/CeVO_4_ (N_5_) nanocomposite with other similar works.

Photocatalyst	Pollutant	Source of light	Radiation time (min)	Maximum destruction (%)	Reference
Polyaniline-modified Fe3O4/SiO2/TiO2	MB	Visible	325	35	(Huang et al., 2011)
Fe_3_O_4_@SiO_2_@TiO_2_@Ho	MO	UV	150	80	(Mortazavi-Derazkola et al., 2017)
Fe_3_O_4_@SiO_2_@TiO_2_–Co/rGO	MB	Visible	160	100	(Fu et al., 2019)
Fe_2_O_3_–Fe_3_O_4_@SiO_2_@TiO_2_–TNS–GR	RhB	Visible	120	93	(Yang and Liu, 2019)
Fe_3_O_4_/SiO_2_/TiO_2_/CeVO_4_	MB	Visible	100	87	This work

### Photo Antibacterial Properties

**Figure 9:**
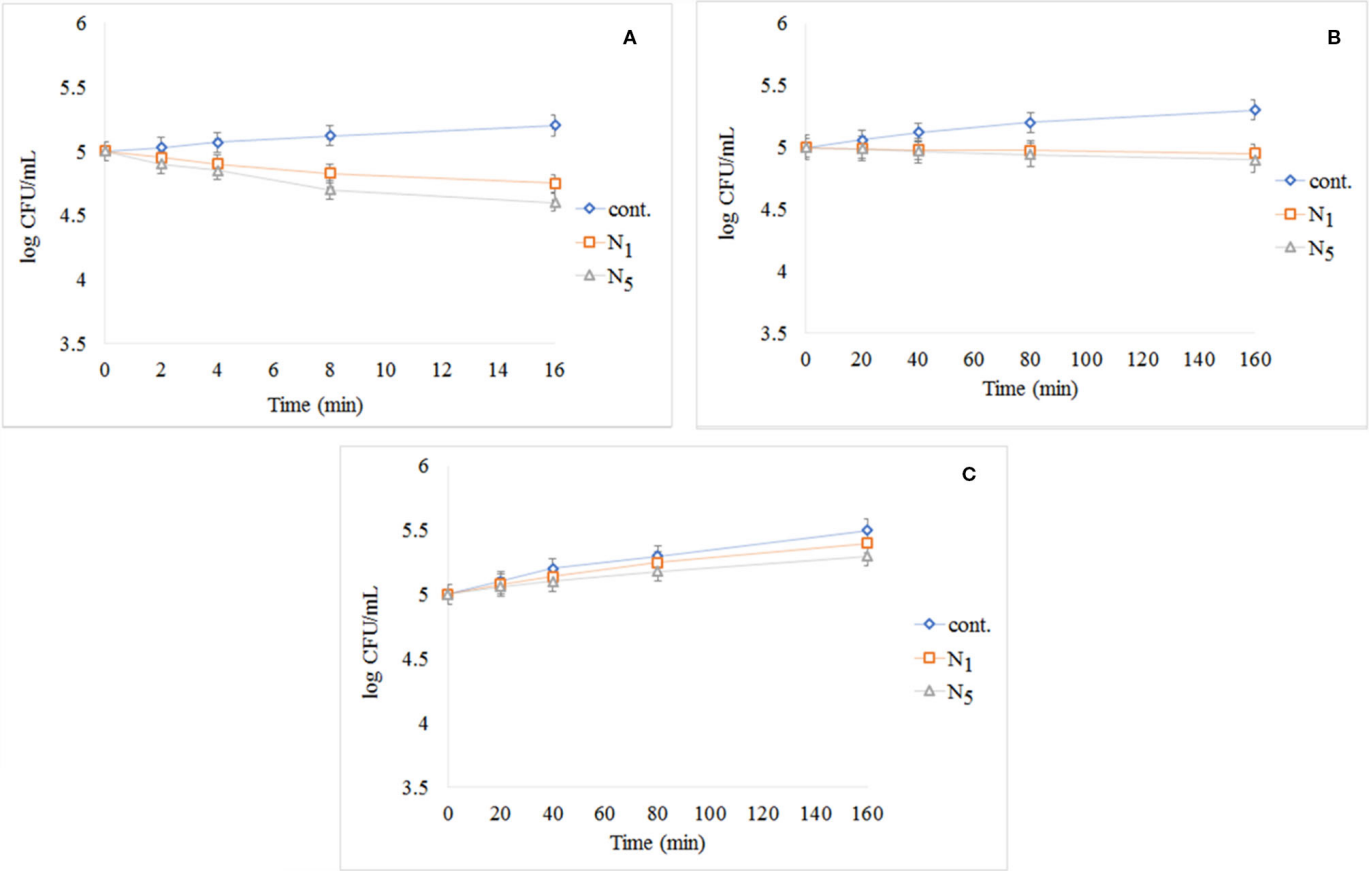
Reduction in cell viability of N_1_ and N_5_ samples for *S. aureus*: **(A)** *S. aureus* exposed to UV light, **(B)** *S. aureus* after exposure to visible light, and **(C)** *S. aureus* in darkness.

**Figure 9A** shows the results of the test performed using *S. aureus* exposed to UV light. The cont. group had a small growth in comparison with N_1_ and N_5_ samples, and N_5_ sample had better performance. The results of the test performed using *S. aureus* exposed to visible light and dark condition respectively as illustrated in **Figures 9B, C**. As shown in **Figure 9B**, the control group has significantly increased by passing the time, but N_1_ and N_5_ samples have prevented the growth of bacteria. Also, seeing the growth for all samples in dark conditions demonstrates that the synthesized samples are light-dependent to take advantage of antibacterial properties.

**Figure 10:**
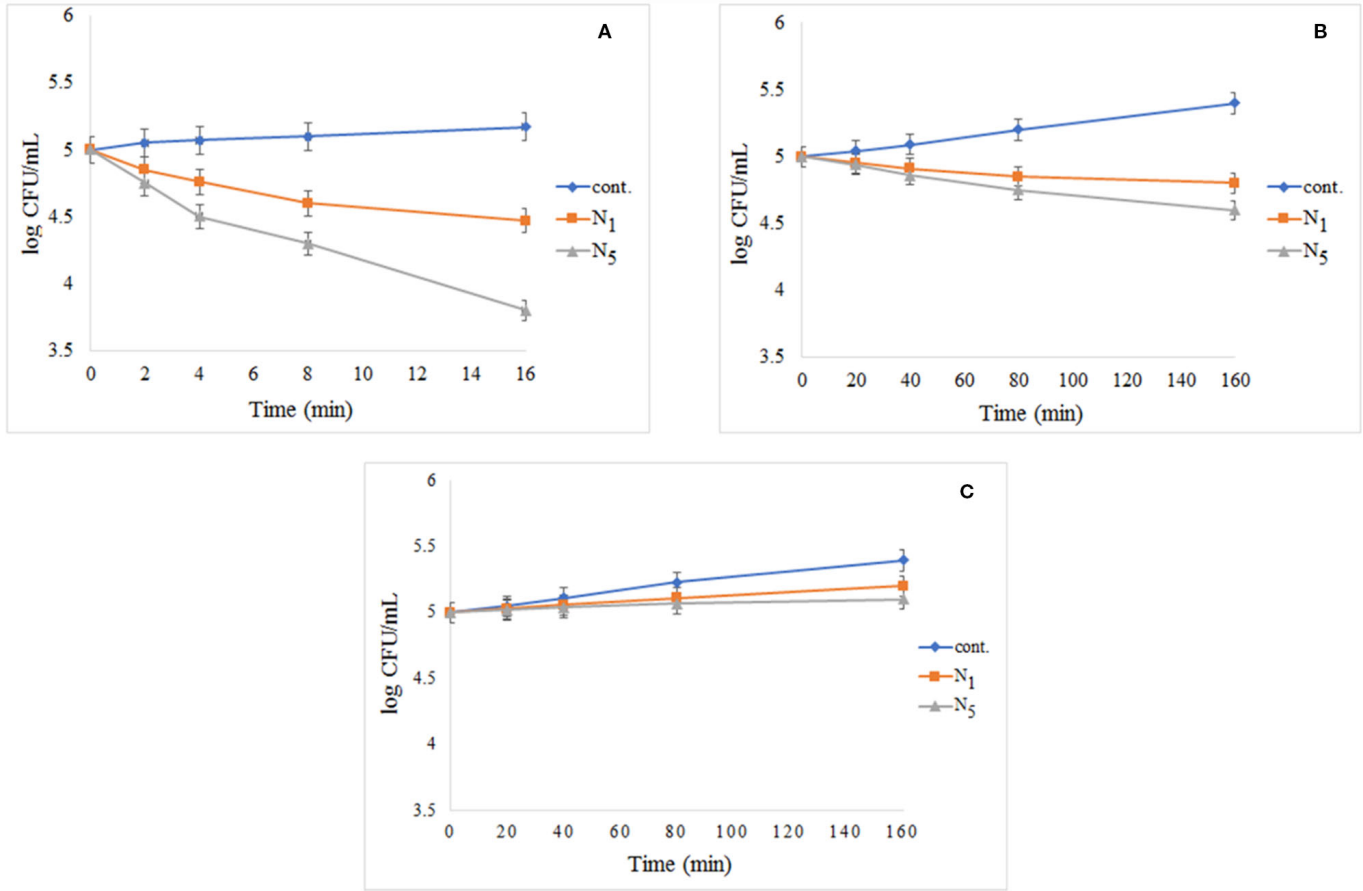
Reduction in cell viability of N_1_ and N_5_ samples for *E. coli*: **(A)** *E. coli* exposed to UV light, **(B)** *E. coli* after exposure to visible light, and **(C)** *E. coli* without light access.

The results of photo-antibacterial examination using *E. coli* has been shown in **Figure 10** by exposing to UV light (a), visible light (b), and dark condition (c). As can be seen in **Figure 10A**, the most decreased in the colony count is related to N_5_ sample nanocomposite and there is no significant change in the control group observed during the test. The decrease of the bacteria was significant in comparison to cont. group. In **Figure 10B**, it was found that the cont. the group experienced significant growth under visible light by during the test, while the N_1_ and N_5_ nanocomposite decreased in compared to the cont. group and the N_5_ sample showed the highest decrease. In **Figure 10C**, we found that all of the samples had experienced growth in darkness as time passes. This issue highlights the importance of light in the reaction system. As presented in the **Figure 10A**, the addition of a third phase (CeVO_4_) to the nanocomposite has led to the elimination of more microorganisms.

### Photodegradation Mechanism

**Figure 11:**
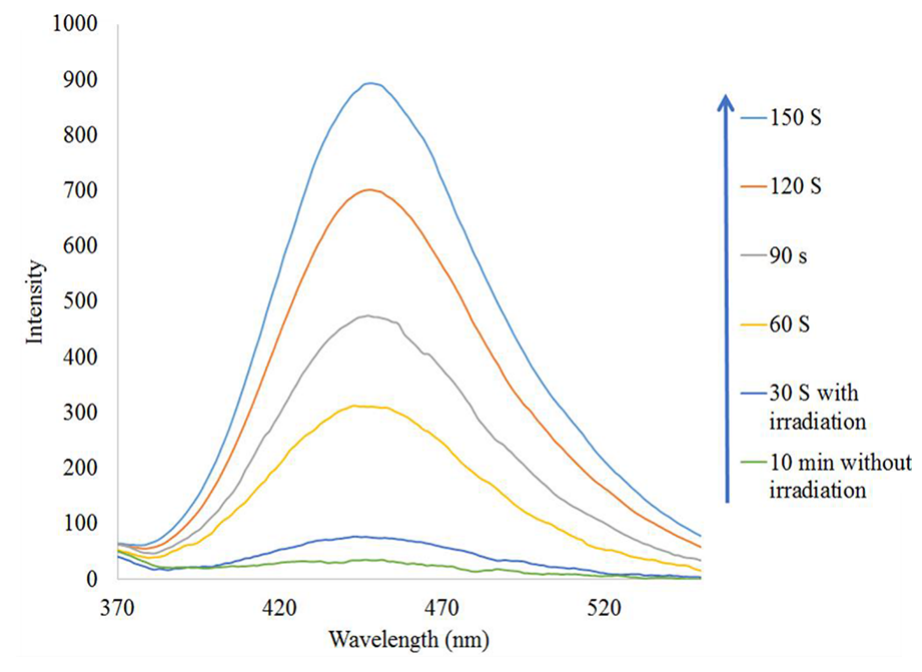
^•^OH trapping PL spectra of Fe_3_O_4_/SiO_2/_TiO_2_/CeVO_4_ (N_5_) nanocomposite with TA solution under UV irradiation.

To prove the presence of ^•^OH in the Fe_3_O_4_/SiO_2/_TiO_2_/CeVO_4_ (N_5_) system during UV irradiation, ^•^OH trapping PL experiments using terephthalic acid (TA) as a probe molecule were also carried out (**Figure 11**). As shown in **Figure 10B**, after irradiation for 150 s, the strong PL signal is seen at 425 nm, and the intensity significantly increased with the irradiation time as a comparison with dark treatment, which shows ^•^OH radicals have been generated.

**Figure 12:**
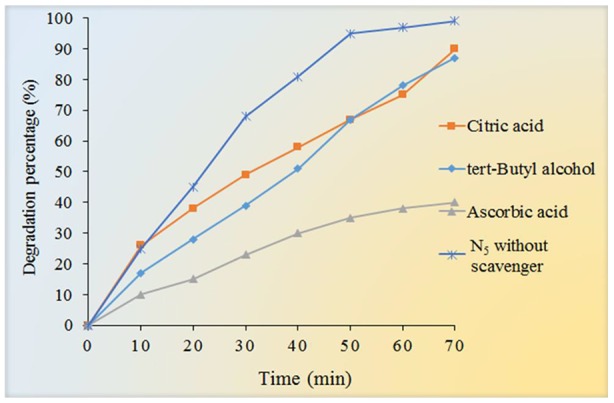
Effect of different scavengers (tert-Butyl alcohol, citric acid, and Ascorbic acid) on the photocatalytic degradation of MB under UV light irradiation.

To study the photocatalytic degradation mechanism of Fe_3_O_4_/SiO_2/_TiO_2_/CeVO_4_ nanocomposite, several agents were utilized to quench the relevant active species within the photocatalytic process, and the results are shown in **Figure 12**. In this study, t-butyl alcohol, Citric acid, and ascorbic acid were adopted to be the scavengers of hydroxyl radicals (^•^OH), holes (h^+^) and superoxide radical (^•^O2^−^), respectively. As shown in **Figure 12**, when t-butyl alcohol and citric acid were added to the reaction system, the photocatalytic efficiency was not noticeably changed but with the use of ascorbic acid, the reaction speed dropped sharply. However, when ascorbic acid was added to the reaction system, the photocatalytic efficiency was reduced from 99.9% to 38% for MB. Based on the results, we can conclude that ^•^O2^−^ is the major reactive species in the photodegradation of MB in the reaction system.

### Cytotoxicity Effect

**Figure 13:**
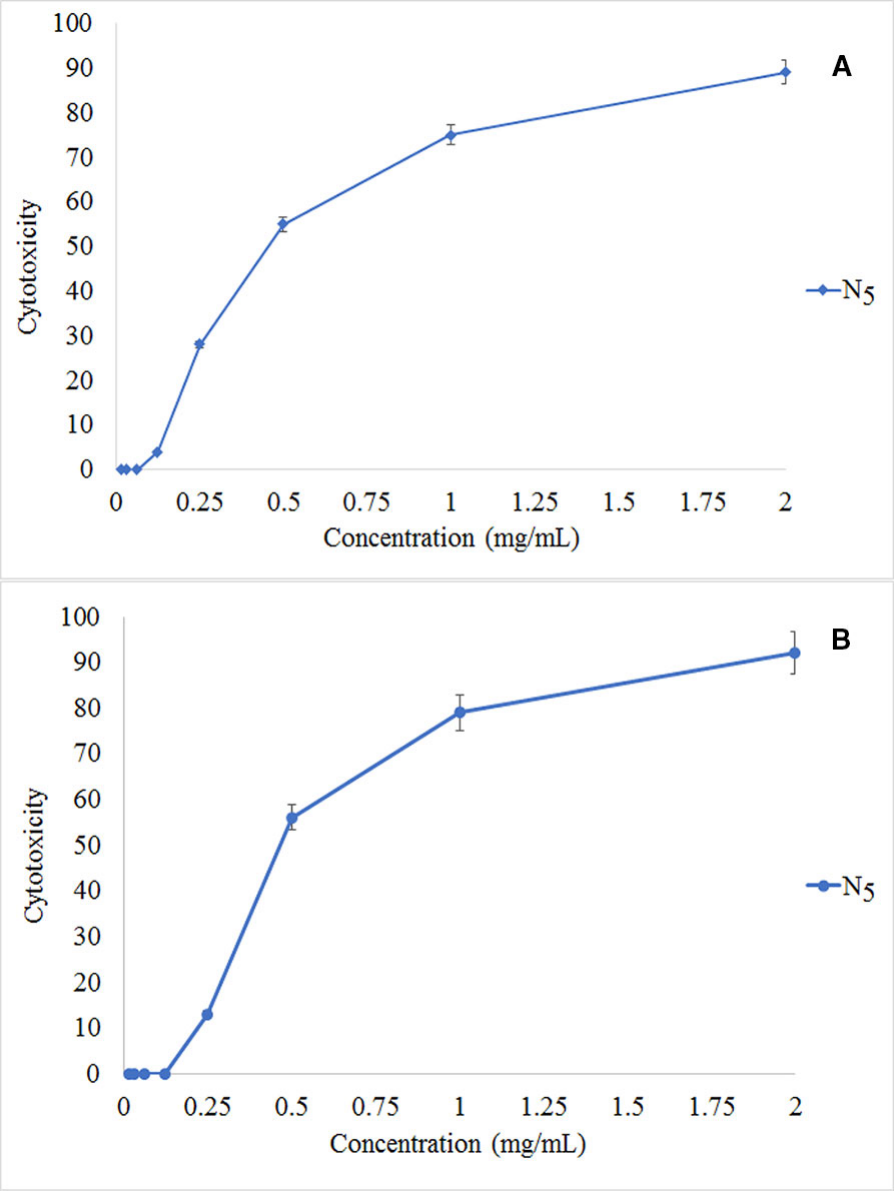
Relative *in vitro* cell viability of optimized Fe_3_O_4_/SiO_2/_TiO_2_/CeVO_4_, MTT assay. Panc1 cells incubated with Fe_3_O_4_/SiO_2/_TiO_2_/CeVO_4_ at different concentration for 24 h **(A)** and 48 h **(B)**.

**Figure 14:**
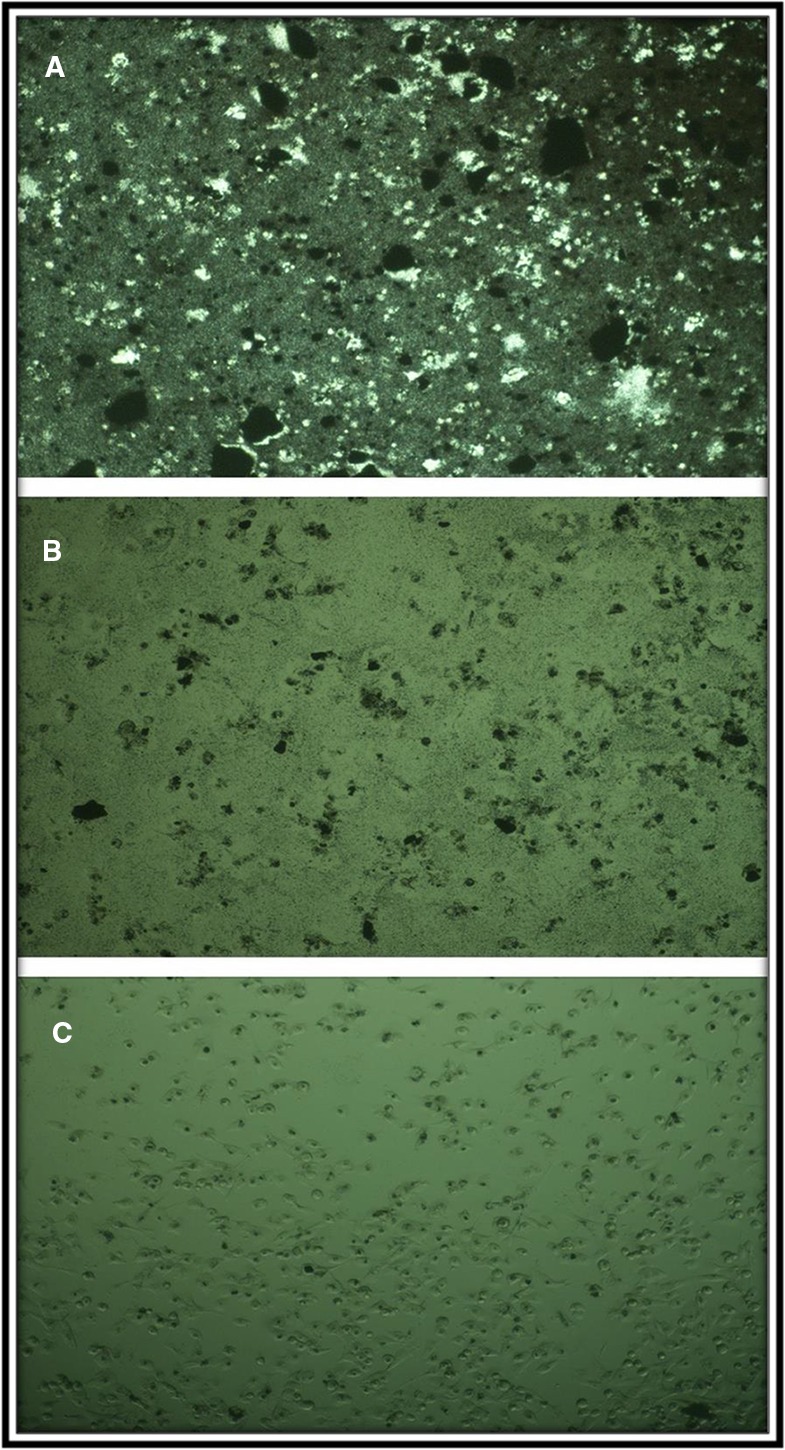
Microscopic photograph of panc1 cell in the presence of C4 specimen at three concentrations **(A)** 2, **(B)** 0.5, and **(C)** 0.0157 mg/ml.

The MTT assay shows that C_4_ nanocomposite had a toxic effect on a panc1 Cell line in a dose-depended manner and its IC50 is about 500 mg/L (**Figure 13**). The concentrations of N_5_ sample used for photocatalytic properties are much lower than that of having toxic effects on mammalian cells. On the other hand, the time taken for the photocatalytic process was at most 160 min, while the effects of cytotoxicity have been investigated after 24 and 48 h. Also, **Figure 14** exhibits the microscopic photos of panc1 cells with optimized nanocomposite (N_5_) at three different concentrations which confirm that once concentrations decrease, the toxicity of the N_5_ sample is reduced as well.

## Conclusions

**Scheme 1:**
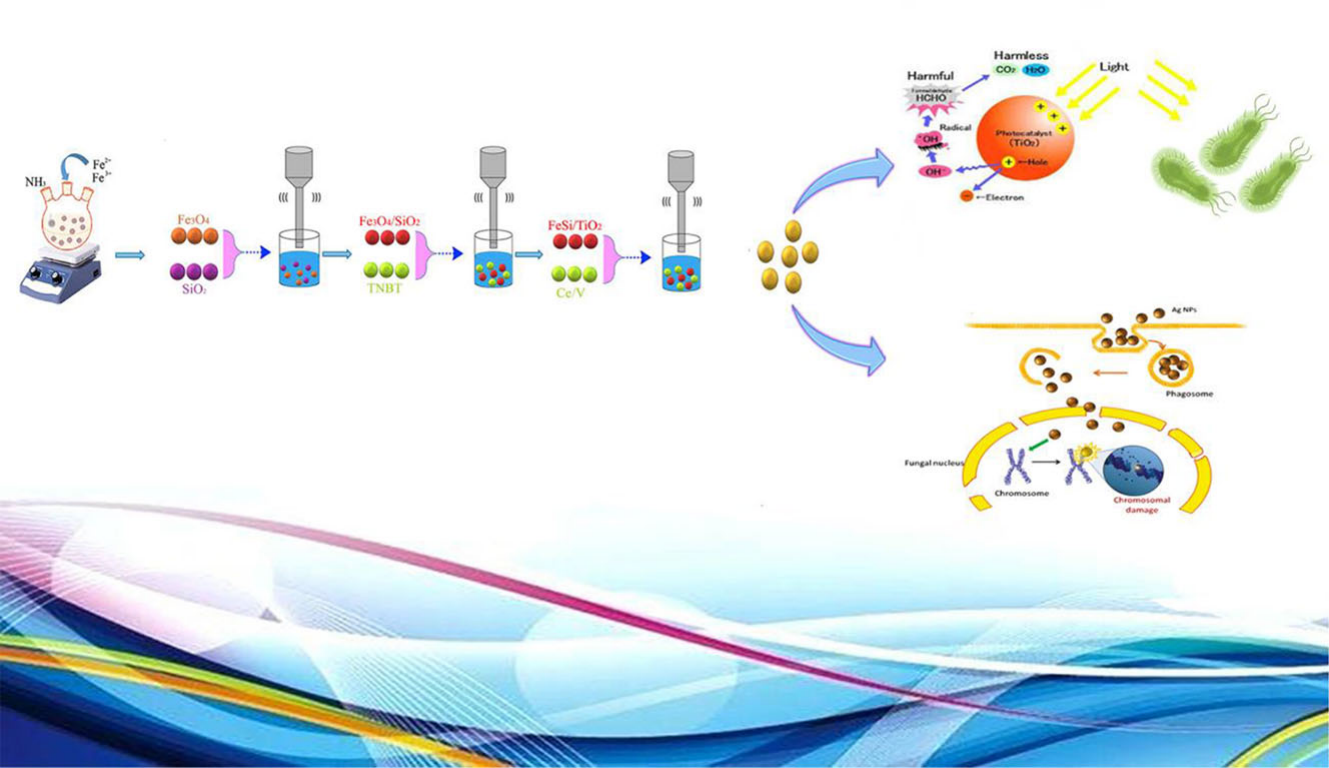
Schematic mechanism for the synthesis of Fe_3_O_4_/SiO_2_/TiO_2_/CeVO_4_ and its different application.

Fe_3_O_4_/SiO_2/_TiO_2_ and Fe_3_O_4_/SiO_2/_TiO_2_/CeVO_4_ nanocomposites were successfully synthesized in different molar ratios by Co-precipitation approach (assisted by ultrasonic method). Also, Schematic mechanism for the synthesis of Fe_3_O_4_/SiO_2_/TiO_2_/CeVO_4_ and its different application is shown in **Scheme 1**. XRD, EDS, SEM, and FTIR methods confirmed the quality and presence of these nanocomposites. DRS data showed a significant decrease in the band gap by adding CeVO_4_ to previous phases. From the photocatalytic test, it was found that the Fe_3_O_4_/SiO_2/_TiO_2_/CeVO_4_ 1:1:1:2 is the optimized sample by photodegradation of methylene blue (∼90% under visible light in 100 min). The photo-antibacterial studies on *E. coli* and *S. aureus* by Fe_3_O_4_/SiO_2/_TiO_2_ and Fe_3_O_4_/SiO_2/_TiO_2_/CeVO_4_ under dark conditions, UV, and visible light irradiation showed that the optimized nanocomposite had better efficiency than Fe_3_O_4_/SiO_2/_TiO_2_ and the cytotoxicity properties of the optimized sample were measured as well. In addition, it was found that the toxic effect on panc1 Cell line is in a dose-depended manner and its IC50 is approximately 500 mg/L.

## Data Availability Statement

The raw data supporting the conclusions of this article will be made available by the authors, without undue reservation, to any qualified researcher.

## Author Contributions

MM and AS contributed in conception, design, statistical analysis and drafting of the manuscript. MR-M, MF-R, KA, ME-A, FA, ES, SM, MG, HE, and YJ contributed in data collection and manuscript drafting. All authors approved the final version for submission.

## Conflict of Interest

The authors declare that the research was conducted in the absence of any commercial or financial relationships that could be construed as a potential conflict of interest.
